# Preparing Data for Artificial Intelligence in Pathology with Clinical-Grade Performance

**DOI:** 10.3390/diagnostics13193115

**Published:** 2023-10-03

**Authors:** Yuanqing Yang, Kai Sun, Yanhua Gao, Kuansong Wang, Gang Yu

**Affiliations:** 1 Department of Biomedical Engineering, School of Basic Medical Sciences, Central South University, Changsha 410013, China; yangyuanqng@163.com (Y.Y.); sunkai16@csu.edu.cn (K.S.); 2 Department of Biomedical Engineering, School of Medical, Tsinghua University, Beijing 100084, China; 3 Furong Laboratory, Changsha 410013, China; 4 Department of Ultrasound, Shaanxi Provincial People’s Hospital, Xi’an 710068, China; gao.yanhua@126.com; 5 Department of Pathology, School of Basic Medical Sciences, Central South University, Changsha 410013, China; wangks001@csu.edu.cn; 6 Department of Pathology, Xiangya Hospital, Central South University, Changsha 410013, China

**Keywords:** artificial intelligence in pathology, data preparation, clinical-grade, deep learning

## Abstract

The pathology is decisive for disease diagnosis but relies heavily on experienced pathologists. In recent years, there has been growing interest in the use of artificial intelligence in pathology (AIP) to enhance diagnostic accuracy and efficiency. However, the impressive performance of deep learning-based AIP in laboratory settings often proves challenging to replicate in clinical practice. As the data preparation is important for AIP, the paper has reviewed AIP-related studies in the PubMed database published from January 2017 to February 2022, and 118 studies were included. An in-depth analysis of data preparation methods is conducted, encompassing the acquisition of pathological tissue slides, data cleaning, screening, and subsequent digitization. Expert review, image annotation, dataset division for model training and validation are also discussed. Furthermore, we delve into the reasons behind the challenges in reproducing the high performance of AIP in clinical settings and present effective strategies to enhance AIP’s clinical performance. The robustness of AIP depends on a randomized collection of representative disease slides, incorporating rigorous quality control and screening, correction of digital discrepancies, reasonable annotation, and sufficient data volume. Digital pathology is fundamental in clinical-grade AIP, and the techniques of data standardization and weakly supervised learning methods based on whole slide image (WSI) are effective ways to overcome obstacles of performance reproduction. The key to performance reproducibility lies in having representative data, an adequate amount of labeling, and ensuring consistency across multiple centers. Digital pathology for clinical diagnosis, data standardization and the technique of WSI-based weakly supervised learning will hopefully build clinical-grade AIP.

## 1. Introduction

Pathological diagnosis stands as the gold standard in disease diagnosis [1], relying on microscopic examination of tissues and cells from glass slides. However, the traditional glass slide format presents challenges in terms of storage, sharing and remote consultation. The advancement of digitalization technology has introduced a transformative solution by enabling the conversion of glass slides into high-resolution whole slide images (WSI) [2] through pathological scanners. This paradigm shift towards digital image-based pathology, often referred to as digital pathology (DP), has gained increasing prominence [3,4].

Recently, the combination of DP and artificial intelligence (AI) has given birth to the new computational pathology (CPATH) or AI in pathology (AIP). This fusion of technologies has shown remarkable potential in enhancing the efficiency and accuracy of disease diagnosis, effectively addressing the scarcity of pathologists. For example, the current shortage of pathologists in China is up to 100,000; in addition, the number of pathologists in the United States also decreased by 17.53% from 2007 to 2017 [5]. In the past few years, a large number of AIP systems have emerged, focusing on tasks such as classification, grading, outcome prediction, prognosis determination [6,7] and the diagnosis of various cancers such as gastric cancer [8,9], prostate cancer [10,11,12,13], bowel cancer [14], breast cancer [15,16,17,18,19], and cervical cancer [20,21] among others.

AIP predominantly relies on deep learning, utilizing datasets consisting of hundreds to tens of thousands of WSIs for training and testing. Although the AIP has proved to be effective and robust, showing extremely high performance, even comparable to pathologists, their high performance is generally difficult to reproduce in the clinic. The pivotal factor influencing the accuracy and generalization capabilities of deep learning-based AI is the meticulous preparation of data, making it a crucial element in surmounting the hurdles faced by AIP when transitioning into clinical practice [22].

Given the extreme importance of datasets to develop effective AI, we conducted a comprehensive review of AIP studies to date, with a focus on data preparation methods aimed at enhancing the accuracy and robustness of AI systems. Our primary objective is to find a solution that can address the impediments to the clinical application of AIP, that is, how to prepare data for developing clinical-grade AIP.

**Figure 1:**
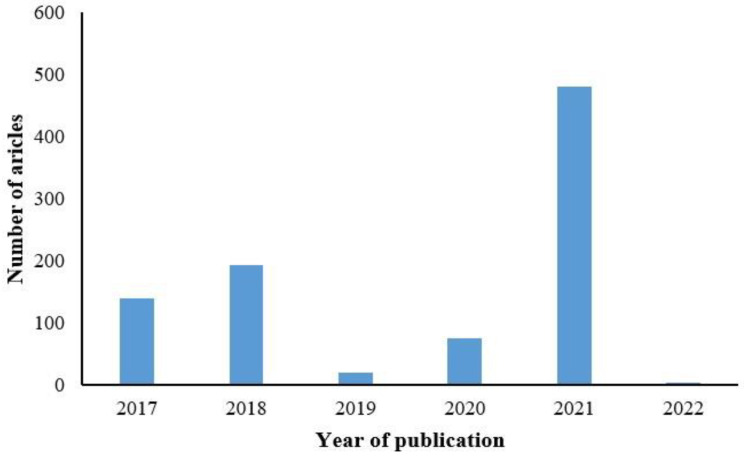
Publications of AIP by years.

Our search encompassed the PubMed database with the time frame set from January 2017 to February 2022, utilizing keywords such as pathology, machine learning, digital pathology, pathological diagnosis, and deep learning. A total of 829 papers were retrieved. Figure 1 illustrates the distribution of AIP studies using deep learning methods over the past five years, with the highest number of 450 studies published in 2021, as depicted in Figure 1.

We initially reviewed paper abstracts, reviewed research types and topics, and identified 220 papers related to pathological images. Following a thorough examination of the full text, we selected 118 papers that align with the objectives of our data preparation analysis, adhering to the following three exclusion criteria.

1. Papers utilizing non-pathological image data were excluded.
2. Works lacking detailed descriptions of the data preparation process or relying solely on public datasets were excluded.
3. Studies exclusively focused on AI or pathology without addressing the intersection were excluded.

**Figure 2:**
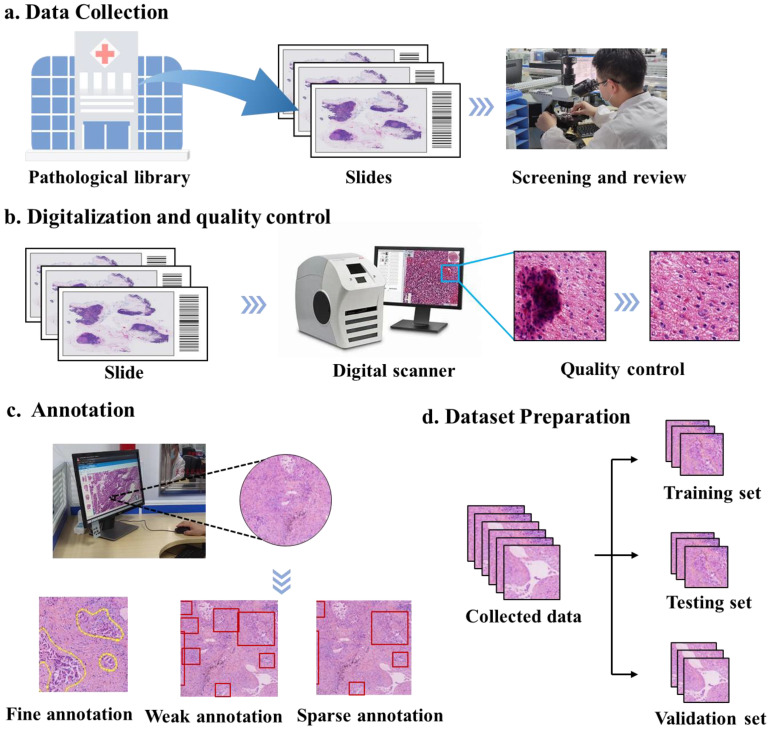
Data preparation pipeline for a robust AI system. (**a**) Data collection. Locating slides in pathological library, followed by a rigorous screening and review process. (**b**) Digitalization and quality control. Digitalizing slides using a digital scanner to obtain WSIs. Employing technology to rectify image distortion. Then pathologists review the quality of WSIs. (**c**) Annotation. This figure illustrates three types of annotation, from left to right: fine, weak, sparse annotation. (**d**) Dataset preparation. The collected data should be divided into training set, testing set, and validation set.

We conducted an in-depth analysis of the 118 selected papers, elucidating the pipeline of data preparation for constructing effective AIP systems, as illustrated in Figure 2, and summarizing detailed information about the datasets used in these papers, such as the number of slides, scanner, patch size and more in the Supplementary Table S1. The first step is to collect tissue samples according to the AIP objectives. Subsequently, meticulous examination is carried out to eliminate dust particles from slides, ensuring the integrity and color fidelity of the tissue. These slides undergo digitization through pathology scanners, resulting in the acquisition of high-resolution whole slide images (WSIs). Quality control measures are indispensable for WSIs to identify and exclude image artifacts, and various annotation methods are used to establish connections between the data and medical facts. After model training, comprehensive validation is essential to assess the accuracy and generalization capabilities of the AIP system.

## 2. Data Collection

### 2.1. Pathological Slide Cellection

AIP relies heavily on the availability of a substantial quantity of high-quality WSIs from clinical diagnoses [23]. However, the tissue specimens are initially preserved in the form of glass slides. Researchers undertake the task of identifying relevant keywords. Subsequently, technicians use these keywords to access patient identities through the Pathology Information System (PIS) and obtain the necessary slides.

The performance of deep learning is heavily dependent on the volume of training data. For example, a substantial increase in the colorectal cancer dataset, expanding from 420 [24] to 13,111 slides [25], resulted in a noteworthy enhancement in AIP accuracy and good multicenter generalization ability [26,27,28]. Impressively, a collection of 44,732 slides from 15,178 cases achieved an impressive area under the curve (AUC) of up to 0.98, even in independent medical centers [23]. Beyond quantity, the representation of various disease subtypes and grades within the slides is equally critical to prevent data bias [28].

Ensuring a balanced distribution of slides across various classes is another critical consideration [20]. Some diseases are relatively rare, thus resulting in a scarcity of slides. An imbalanced dataset, as observed in renal cell carcinoma, led to a 10% lower recall rate for disease types with limited sample sizes compared to the more prevalent subtypes [29]. When factors such as age, gender, and post-surgery outcomes potentially impact results, it is imperative to ensure an equitable distribution of slides for each factor. One approach to maintaining data balance is to randomly select a subset from classes with large sample size. For example, in order to equalize the number of survivors and deaths of colorectal cancer patients within five years after surgery, 182 survivor data were randomly removed [24]. Increasing the number of slides for classes with small sample sizes from other centers is also a viable option.

To mitigate the influence of uncontrollable factors, the collection of slides should be as randomized as possible [14]. For example, reagent types and preparation methods may exhibit temporal variations. Therefore, the timespan during which slides were produced should be as extensive as possible to encompass variations in the production process. For instance, collecting 250 specimens from January 2009 to December 2017 resulted in an AUC of 0.95 for a hepatocellular carcinoma prognostic model [30]. Similarly, as the span of disease-free survival and age were as long as 4–86 months and 25–75 years respectively, the prognostic model of oral squamous cell carcinoma achieved high accuracy up to 96.31% [31]. However, it is worth noting that extensive assessments of potentially influential factors such as ethnicity and brand of tableting drug are still lacking.

Collecting slides from multiple centers [8,22,32,33,34,35], each with distinct production protocols and drug usage, is a recommended practice. AIP trained on one data from one center may suffer from a significant performance degradation when applied to other centers [36]. Multi-center training datasets can help mitigate this issue. For instance, a model trained on data from multiple centers has an average Dice coefficient of 5.6% [22]. Combining slides from three hospitals with different production protocols and four pathological scanners improved the model’s AUC from 0.808 to 0.983 [20]. In addition, slides from multiple centers allow for a more comprehensive generalization assessment, as discussed in Section 6.

Public datasets like the TCGA (The Cancer Genome Atlas) can serve as valuable supplements. Many AIPs are trained and tested by public datasets [16,25,26,30,37,38,39,40], and there are also some challenges that provide pathological images, such as the Grand-challenge (https://camelyon.grand-challenge.org, accessed on 15 June 2022) [41], MITOS-ATYPIA [42] etc. listed in Table 1.

However, the images in public datasets are usually derived from a small number of slides. For example, the PAIP dataset contains only one hundred WSIs. Second, the staining process of the same WSI is similar, so the patches cut from the same WSI are also similar. Third, the public datasets may be only applicable to specific diseases, for example, the GlaS contains only images of T3 or T4 colorectal adenocarcinoma.

### 2.2. Ethics Statement

The ethical approval from the local ethics committee is a fundamental prerequisite for AIP studies. Although most studies are retrospective and do not necessitate informed consent from patients [9,24,48], however, for some prospective studies [11] such as disease outcomes [31,49], private information (name, date of birth and so on) should be anonymized 16. The National Management Measures for Health Care Big Data Standards, Security and Services (Trial) [50], Health Insurance Portability and Accountability Act (HIPAA) [51] and other related laws should be complied strictly in data collection, storage, usage and disclosure.

### 2.3. Slide Screening and Review

Given the extended storage duration of many slides, a thorough cleaning process is essential to eliminate any contamination. Maintaining consistent high-quality production for a large number of tissue specimens can be challenging. After cleaning, the quality of the slides must be carefully checked under microscope. This assessment includes scrutinizing the integrity of tissue specimens, detecting tissue folding, identifying air bubbles, evaluating staining quality, and checking for any signs of fading [33]. 

In cases where the specimens were folded and wrinkled, addressing these issues is critical. Studies have shown that when dealing with such unaddressed anomalies, the mean absolute error between the immunohistochemical score calculated by the model and the results predicted by the pathologist was 2.24 higher [52]. Consequently, unqualified slides should be excluded before the next. In addition, since misdiagnosis always happened, it is essential to ensure the diagnostic accuracy of the collected slides [32]. As a precaution, a review of the slides, either at present or following digitization, is recommended to maintain data integrity and accuracy.

## 3. Digitalization and Quality Control

### 3.1. Digitization

High-resolution WSIs are obtained from slides through the use of a fully automatic pathological scanner [53], providing a wealth of information about the morphological and functional characteristics of biological systems [54,55]. WSIs are available in various file formats, including KFB file from Ningbo Jiangfeng Bio-Information Technology Co. (KFBIO company, Ningbo City, China) [32], Leica’s SVS file [30] and TIFF file [23], causing some trouble for data sharing [56]. To mitigate this issue, files should be converted into universally compatible image formats such as JPEG by the software library of manufacturer.

As the production of pathological scanners by different manufacturers continues to grow, disparities in the resulting WSIs inevitably emerge [57]. These variations can have a notable impact on the performance of AIP. For example, when prostate cancer slides initially scanned with an Olympus VS120-S5 were subsequently rescanned using a Philips Ultra-Fast scanner, there was a notable 5% increase in the Area Under the Curve (AUC) [58]. 

**Figure 3:**
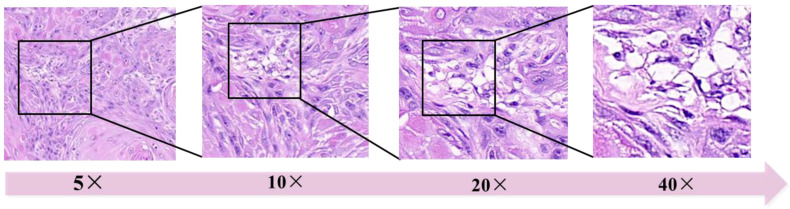
Different magnifications of WSIs (5×, 10×, 20× to 40× from left to right).

The magnification of WSI has a significant impact on model performance [59]. While scanning slides with lower magnification such as 5× (times), may not readily reveal cellular morphology but provide a macroscopic view of tissue structure. Conversely, higher magnifications yield finer details but entail the inclusion of more redundant pixels, leading to large amount of computation to AI model (Figure 3). Usually, 20× [25,60,61] and 40× [20,30,37,62] WSIs are used for most AIP. 

To overcome the differences of scanners during digitization, recent studies have highlighted the importance of employing a diverse array of scanners [7,16,20]. This approach helps minimize the impact of differences in sharpness, resolution, and imaging differences on AIP. The magnification, color fidelity and imaging quality of scanners should be carefully evaluated when selecting scanners. Notably, the Digital Pathology Commission of the Federal Association of German Pathologists has developed a guideline for pathology digitization [63], delineating the minimum technical requirements for scanner systems that can be used in digitalization. However, the absence of overarching standards and specifications underscores the pressing need for a more robust and universally calibrated evaluation system to uphold scanner validity.

### 3.2. Post-Processing after Digitalization

The quality of some WSIs may not be ideal for developing artificial intelligence. The tissue folding, poor staining and other problems in slides may be introduced into the images [8]. The digitalization may encounter challenges, such as defocusing [53]. The main distortion in WSI lies in color change, partial out-of-focus and noises. Studies have shown that the accuracy of AIP was reduced by 6–22.8% on the images without image normalization [64]. Therefore, post-processing after digitalization is necessary to improve performance.

#### 3.2.1. Color Normalization

**Figure 4:**
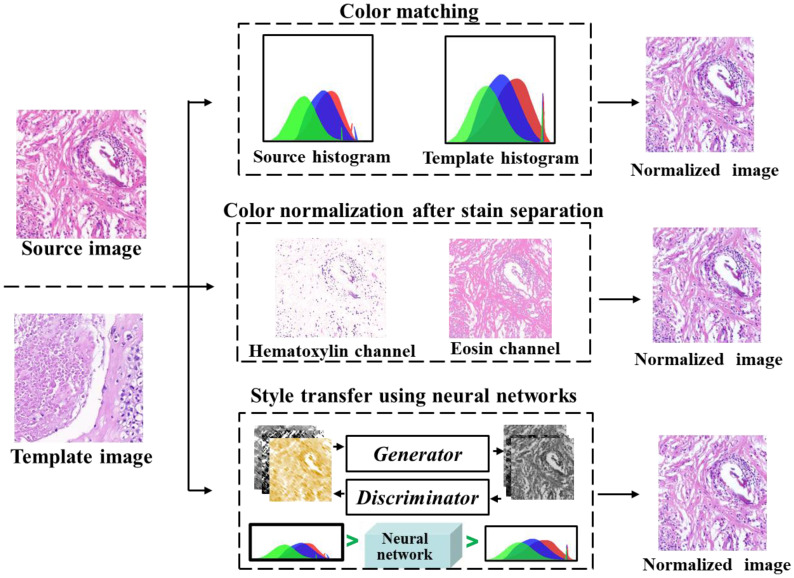
Three methods of color normalization. From top to bottom: Histogram-Based Color Matching (Red Box): This method involves color normalization using histogram-based matching. Color Normalization After Stain Separation (Blue Box): Here, the contribution of individual stains is separated, and the input image is aligned with the template image. Style Transfer Method (Green Box): This technique transforms the color style of the source image to match that of the template image.

Various methods for color normalization have been proposed, including color matching, color normalization after stain separation, and neural networks for style transfer, as illustrated in Figure 4. The color matching aligns the statistical color and intensity distributions (e.g., mean and standard deviation) between a source image and a pre-selected target image [65], where the histogram specification is the most commonly used [66]. However, this method uses contrast stretching forcing the histogram of source image to match the histogram of destination image, resulting in unnatural effects [67], which may lead to unnecessary bias to subsequent image analysis [68].

The staining separation method normalizes each color channel individually. Due to the non-linear relationship between the concentration of RGB dyes and light intensity, direct use of RGB for dye separation is not feasible. Therefore, the RGB channels are converted to optical density (OD) space, where dye concentration and light intensity is linearly separable [67]. The image intensity (*V*) is defined as the logarithm of the ratio of incident ($I_{0}$) to transmitted light intensity (I):

(1) $$V=log10\left(\frac{I_{0}}{I}\right)=W\cdot H\;$$

The OD value is the staining vector (W) times the staining density map (H). Recent studies have employed neural networks to automatically estimate the appropriate W and subsequently conduct a deconvolution operation for image reconstruction [69,70,71,72].

Color normalization has evolved into a technique known as style transfer [73]. A generative network is employed to adapt the input image to the color style of a target image, effectively restoring normal color features [64]. Importantly, this method achieves similarity between the input image and the target image without requiring a reference image. A more recent advancement involves the use of conditional generative adversarial networks (cGAN) for color normalization. This approach reduces the reliance on manual selection and overcomes the limitation of learning a single-color style, as observed in prior studies [74].

#### 3.2.2. Image Distortion Correction

**Figure 5:**
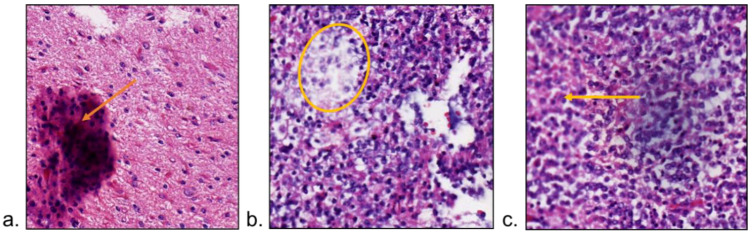
Artifacts in histopathology images. (**a**) Typical Tissue Folding (Yellow Arrow): In this diagram, folded tissue, as indicated by the yellow arrow, appears thicker than the surrounding tissue. (**b**,**c**) shows regions out-of-focus: these regions are highlighted by the yellow circle and arrow, respectively, indicating areas where the image is out of focus.

The presence of bubbles and tissue folding in slides will lead to artifacts in WSIs [75]. The saturation changes in the folded tissue (Figure 5a) may degrade the AI performance. The folded regions can be detected by enhanced brightness of image pixels, but some isolated pixels may be mislabeled [76]. If the connectivity of saturation and intensity was used for folding detection in low resolution WSI, AUC improved by 5% after excluding the regions with tissue folding [77].

The higher magnification lens in the scanner processes a narrower depth of field, making the WSI susceptible to going out-of-focus (Figure 5b,c) when dealing with uneven tissue on the slide, resulting in blurred images. Although imaging algorithms strive to adjust focus positions, limitations in scan speed often result in localized blurring [78]. Such blurriness can adversely impact the detection and classification of certain diseases, where the out-of-focus normal tissue may be misjudged as the tumor [79].

To address this challenge, a detection method for detecting blurred regions was proposed based on local pixel-level metrics. This innovative approach achieved an AUC surpassing 0.95 [80]. Furthermore, a convolutional neural network was trained to serve as a blur detector, effectively reducing detection errors by 12.3% [81]. However, there remains a dearth of out-of-focus correction methods for WSI. A promising development involved the proposal of a deblurring method for pathological microscope images, showcasing the potential for enhancing the clarity of pathological images through post-processing [82].

#### 3.2.3. Data Augmentation

**Figure 6:**
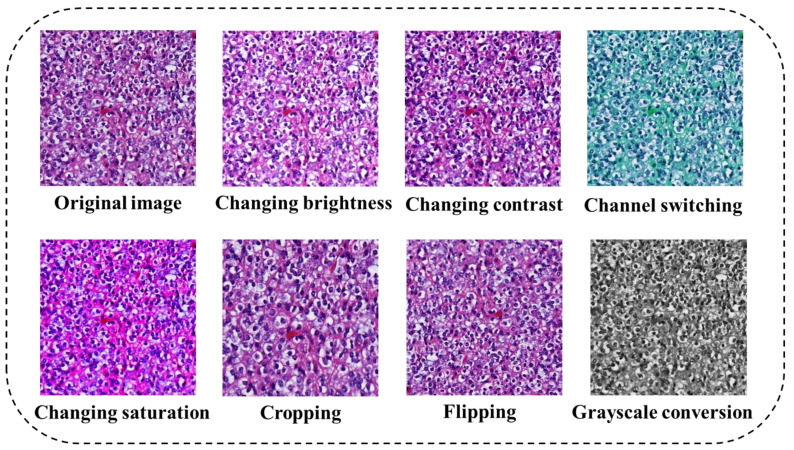
Example images of various data augmentation methods.

Deep learning-based AIP often require extensive quantities of labeled data to achieve high performance [26]. However, labeled data are often difficult to obtain, especially for some rare diseases. Therefore, the data augmentation techniques are widely employed such as cropping, rotating, flipping images [83], changing image contrast and brightness [9] and so on. Example images of various data augmentation methods are shown in Figure 6.

The generative adversarial network (GAN) to synthesize new data has become promising currently [84]. Within this framework, the generator component creates fresh synthetic images while the discriminator distinguishes them [85]. These synthetic images serve to augment sample sizes, especially for rare diseases, potentially enhancing the accuracy of artificial intelligence in pathology. For example, the inclusion of 3000 glioma histopathological images generated by GAN in the training set for predicting the status of the glioma marker isocitrate dehydrogenase, significantly increased the prediction accuracy from 0.794 to 0.853 [86]. The conditional GAN (cGAN) has also been used to augment training data [13], resulting in a remarkable 7% improvement in the classification accuracy of prostate cancer, outperforming the conventional method’s 2% gain.

While synthetic data alleviates the challenge of amassing large labeled datasets, it is crucial to acknowledge that images generated by GANs can exhibit a variety of illusions or artifacts, including checkerboard patterns, blurriness, and excessive smoothing. These issues can result from improper network architecture, inadequately designed loss functions, suboptimal training techniques, and poor-quality training data [87]. Especially for histopathological images, which are replete with intricate structural and texture features [88]. Consequently, GANs operating on such data can be intricate and somewhat unstable. For example, the colorectal cancer images generated by cGAN looked blurry and slightly lost the image details [89]. In-depth evaluation by two pathologists revealed that while GANs were effective in maintaining clear image boundaries and accurate cytoplasmic colors, they still exhibited inaccuracies such as blurred chromatin, a lack of nuclear detail and incorrect texture of keratin flakes [90].

Therefore, despite the visual authenticity of generated images, they should not be included into the dataset without rigorous validation [91,92]. The images without further confirmation may adversely affect data distribution and degrade the model’s performance.

#### 3.2.4. WSI Review

In order to ensure the quality of data, a critical step involves the reevaluation of WSIs before subsequent preparation. Typically, a dual review by two senior and experienced pathologists is needed on each WSI. If the assessments align, this WSI can be included in the dataset, otherwise it will be discarded [25]. Additionally, a thorough check is performed to ensure that WSIs do not exhibit severe color distortions, artifacts, and blurriness. While human review is feasible during the creation of training and testing datasets for AIP. However, in the context of AIP applications in clinical prediction, automatic detection techniques discussed in Section 3.2 should be developed to exclude the WSIs with the presence of severe artifacts, which may cause a significant drop in performance.

#### 3.2.5. Patch Extraction

Given that the image size of 40X WSI can extend up to dimensions of 100,000 × 100,000 pixels, they cannot be directly input into the graphics processing unit (GPU) for training and testing. Therefore, it is common practice to manually or automatically extract small regions of interest (ROIs) related to the objects such as image region of diseases [25,40].

The height or width of the ROI include up to thousands of pixels, which exceeds the default input of most neural networks, prompting further segmentation into some non-overlapping patches. These patches typically take the form of square areas with dimensions ranging from 32 × 32 to 1000 × 1000 pixels [86]. At a magnification of 20×, the 256 × 256 [62,93,94,95] 224×224 [26,74] pixels are common sizes for each patch, matching the input size of most neural networks.

## 4. Annotation

### 4.1. Annotation Methods

Artificial intelligence in Pathology primarily relies on supervised deep learning, thus requiring a substantial volume of accurately annotated data. Image annotation establishes association between images and medical events, such as diagnostic results, which is essential for supervised deep learning. Therefore, a sufficient number of accurately annotated images can enhance system’s accuracy [96,97]. The pathologists routinely employ WSIs for pathological diagnosis, it can be considered that all the WSIs are inherently annotated. However, the WSIs are often immense, and the regions of interest are typically minuscule, resulting in annotations that are not intricately linked to the specific regions. Furthermore, building artificial intelligence systems in pathology directly using WSI-based annotations is often impractical due to computational constraints [98].

**Figure 7:**
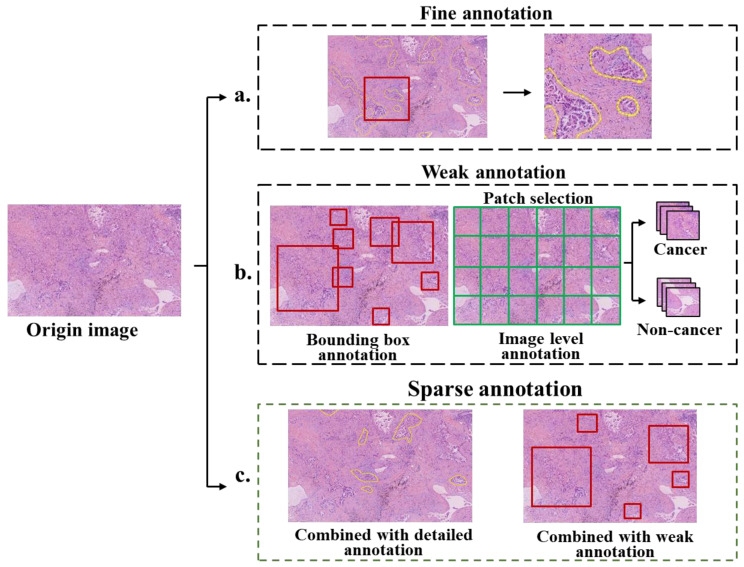
Examples of various annotation methods. (**a**) Fine annotation, where the boundary between the tumor and normal tissue is depicted carefully. (**b**) Left: weak annotation using the bounding box, right: weak annotation using a series of non-lapping patches with labels of cancer and non-cancer. (**c**) Sparse annotation, where only some and not all targets in the image are annotated. From left to right, sparse annotation combined with fine annotation, sparse annotation combined with weak annotation.

**Figure 8:**
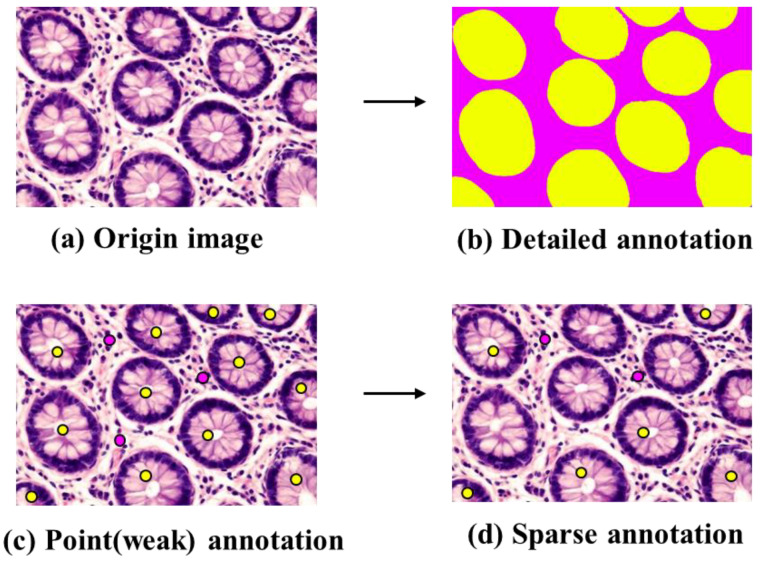
Various annotation methods. (**a**) the original image. (**b**) fine annotation for the segmentation task, in which the yellow regions represent the cells and the purple represents the background. (**c**) weak annotation (point labeling), where the yellow dots represent the location of the cells, while the purple dots represent the background location. (**d**) sparse annotation, where only some cells are annotated by yellow dots.

In order to attain the level of accuracy necessary for robust AI, it is often necessary to further narrow down the annotation, particularly concerning disease location. However, the annotation of WSI is a complex task, and only professional pathologists can determine the accurate locations. At present, the annotation methods can be categorized into three main types: fine annotation, weak annotation and sparse annotation, as shown in Figure 7 and Figure 8.

### 4.2. Fine Annotation

Fine annotation, often referred to as pixel-level annotation, is commonly used especially for image segmentation [47,99,100]. This method involves precisely delineating the location or boundaries of target tissues or cells, effectively connecting individual image pixels with specific targets. For instance, the segmentation of kidney tissue, performed via meticulous pixel-by-pixel fine annotation, achieved a remarkable Dice Coefficient of 0.95 for glomerular segmentation [101]. The colorectal cancer dataset in the DigestPath 2019 challenge was also finely annotated, the proposed segmentation method achieved Dice Coefficient with 0.7789 and AUC with 1 [102].

However, it is important to note that fine annotation is an inefficient and time-consuming process, as it involves precise outlining of boundaries/contours or annotating individual cells. This demanding nature often requires the expertise of multiple experienced pathologists. Notably, boundaries between tissues are often ambiguous, leading to inconsistencies in labeling among pathologists. Due to these challenges, the usage of fine annotation is decreasing in addition to the segmentation or measurement for geometric parameters [23,93,103].

### 4.3. Weak Annotation

In order to alleviate annotation workload, recent approaches have increasingly employed annotations involving bounding boxes [104] and points [103,105] as alternatives to fine annotation. These annotations only point out the target object without necessitating precise location or boundary delineation. Weak annotation strategies have also demonstrated substantial potential in achieving high-performance results. For instance, the classification accuracy of melanoma images labeled with bounding boxes reached 86.2%, outperforming the accuracy of dermatologists at 79.5% [106]. Since bounding boxes for annotation of dense cell or lesion tissue often overlap each other, point annotation is widely used for cell segmentation tasks. On the ISBI Cell Tracking Challenge dataset in 2020, coarse point labels for cell locations yielded an average Dice value of 0.639 [107].

Another prevalent approach involves annotating image patches. WSIs are initially divided into non-overlapping patches with the same size manually or automatically. For example, some patches contain cancer cells, while others exclusively feature normal tissues. Patch-level annotation tends to involve a more manageable workload compared to bounding box or point annotation, significantly enhancing the efficiency of the annotation process [25,59]. In a specific case, benign and malignant hepatocellular carcinoma were labeled at the patch level, resulting in a Dice coefficient of 0.767 for liver cancer cell classification, slightly outperforming model trained on fine annotation data (the Dice was 0.754) [59]. Moreover, the performance of AI frequently benefits from training with large-scale weakly annotated data compared to small-scale fine annotation data [23].

Recently, several studies based on multi-instance learning have embraced WSI-level annotation, where annotations pertain to the entire image without dividing WSIs into smaller sections [108,109]. This approach is advantageous in terms of time and effort efficiency and shows great promise [110]. However, the performance of AI models in pathology relying on WSI-level annotation warrants further evaluation. Many published studies have relied on public rather than clinical datasets. Moreover, the presented models can often achieve good results primarily when the disease area within the WSI is extensive, potentially making them less effective compared to models employing weak annotation at the patch level.

### 4.4. Sparse Annotation

Sparse annotation is a strategy to reduce the annotation workload by labeling only a limited number of objects while leaving a substantial number unlabeled. The sparse annotation is often combined with other annotation methods to reduce overall annotation effort. For example, in combination with weak annotation, sparse annotation was employed to label cells using a limited number of points, resulting in a trained model with 90.1% accuracy and a Dice coefficient of 93.1% [103]. Another example involves the use of sparse and fine annotation to segment gastric tumor images, achieving an intersection over union (IOU) of 0.883, and average accuracy of 0.9109 [96].

## 5. Dataset Preparation

**Figure 9:**
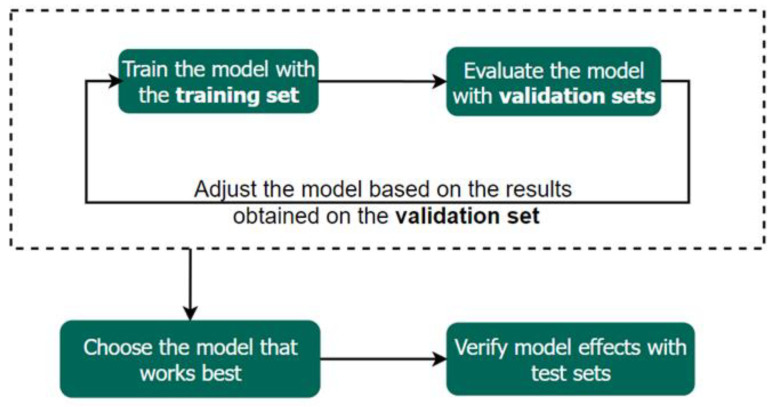
The process of building AI. Initially, the training set is used for model training, followed by the validation set for selecting hyperparameters through the evaluation of model performance. After multiple rounds of validation, the hyperparameters with the best performance are employed for training, and finally, the test set is utilized to assess the AI’s overall performance.

Data collected for AI in pathology is typically divided into three subsets: training set, validation set, and test set [60]. The training set is primarily utilized for generating AI model and constitutes the largest portion of the data. The validation set plays a critical role in model selection, aiding in the selection of hyperparameters that yield optimal results. Finally, the test set is crucial for evaluating the accuracy and overall performance of the model. Figure 9 depicts the role of each dataset in building the model. While specific ratios for these datasets may not always be specified, the configuration of the training set is particularly pivotal in ensuring the robustness of AI in pathology.

(1) To maintain the independence of each set, divisions should be made at the patient level. This ensures that the WSIs from the same patient or patches cut from the same WSI will not appear in different sets [98].

(2) The characteristics of the training set greatly affect the performance of AI. It should cover various disease subtypes and cell morphology distributions, which is close to the real data distribution encountered in clinical practices.

(3) When dealing with a limited dataset, it is advisable to maximize the utilization of WSIs in the training set. For instance, utilizing 205 WSIs out of a collection of 227 WSIs as the training set yielded an 82% accuracy for high-grade ovarian cancer [7]. Conversely, reducing the training set size from 5045 WSIs to 1257 WSIs resulted in a 5.58% decrease in AUC for the classification of phosphorylated cell carcinoma [26]. When data collection is sufficient, the validation set can be expanded accordingly. For example, in a lung cancer dataset comprising 5734 WSIs, 3554 WSIs were designated for training, and 2180 WSIs for evaluation, yielding a AUC exceeding 0.97 [100].

(4) AIP generalization is often assessed by collecting data from multiple different centers. For example, the data from one single center can be served for training, while the data from other centers can be served as an independent test set [14,111]. Some studies incorporated multi-center data into the training set, so that the model can be adaptive to the differences of the production and digitization process. As an example, using data from five centers in the training set for cervical cancer screening achieved a specificity of 93.5%, and a sensitivity of 95.1% in multi-centers [20].

## 6. Limitations and Improvements of Evaluation

Evaluation of AI in pathology is crucial to establish its clinical effectiveness. The evaluation methods for deep learning fall into both internal validation and external validation approaches. When the data available is insufficient, randomly selecting a subset for testing is commonly used; however, it can introduce significant performance fluctuations. Cross-validation is a valuable technique in evaluating the performance [100,112], where the dataset is randomly divided into K mutually exclusive subsets. Each time using one subset for training and one subset for testing, the training and testing are repeated K times respectively. The resulting mean and confidence interval of the K-fold cross-validation can eliminate the randomness effect caused by a single data division, leading to a more reliable assessment.

However, cross-validation, while valuable, remains an internal validation method as it uses data from the same source. Therefore, its ability is confined to evaluating the model’s performance on samples from the same center, potentially leading to overestimations of performance [27,113].

More precise evaluations can be achieved using data entirely independent of the training set [114]. The data from different centers, diverse scanners, or distinct production protocols can better assess the generalization capabilities. For instance, the AUC of the translocation renal cell carcinoma model reached 0.886 in internal validation and 0.894 in an independent external dataset, demonstrating consistency [66]. Similarly, a semi-supervised approach for colorectal cancer recognition achieved an AUC of 0.974 on 12,183 WSIs from 12 medical centers, slightly outperforming pathologists with an AUC of 0.969 [25].

It is worth noting that the testing set is significantly smaller than the clinical WSIs, making it nearly impossible to cover all the cellular and histological patterns present in clinic. Laboratory evaluation, therefore, falls short of addressing the complexities of clinical application. After a blinded study on an external testing set, the misdiagnosis of the presented models still occurred clinically in 17 sites and 6 cases, including misdiagnosis in detection, grading of prostate cancer and detection of perineural invasion [115]. Additionally, some models’ performance experienceda great decline when confronted with datasets from different countries, medical centers, patient populations and even pathological scanners. For example, t breast cancer images from diverse sources and scanners resulted in a 3% decrease in AUC [79]. Therefore, an overly optimistic view of the actual clinical performance of AI in pathology should be avoided [116]. To properly validate the real performance of the model, it is urgent to collect data from different countries [117], and different medical centers to conduct prospective studies [49,113,117,118], thus enhancing model generalization and robustness.

It is important to note that in clinical practice, even within the same healthcare center, variations can occur in both histological and digital processes. For instance, it is challenging to ensure complete consistency in the staining process for each specimen, and aging or color changes in scanners can introduce artifacts into the images. Additionally, nearly all assessments rely on pathologists’ annotations as the ground truth. However, the variability and subjectivity among different pathologists in their assessments suggest that a certain level of inherent uncertainty exists. This inherent uncertainty implies the need for increased caution and complexity in AI assessments to ensure the accuracy and reliability of the results.

The key to deploying AIP in clinical practice lies in its ability to predict data unseen in the training set, with one feasible method being to assess AIP in clinical practice. The evaluation of AI should be prospective and oriented towards practical application in the real world. This entails involving multiple centers and pathologists and taking into account the diversity of clinical pathological conditions to validate the robustness of AI across various target images. Therefore, obtaining external validation data directly from clinical sources or synchronously validating it with pathologists’ diagnostic results is crucial for reliably assessing the repeatability of AI performance. External validation must demonstrate that AIP exhibits high reliability and accuracy, providing real benefits to patients. Hence, there is an urgent need for prospective clinical trial evaluations to demonstrate whether AIP tools can have a positive impact on patients [119].

There are already some guidelines and standards to aid in the assessment of AI in pathology. The Standard Protocol Items: Recommendations for Interventional Trials-Artificial Intelligence (SPIRIT-AI) and Consolidated Standards of Reporting Trials-Artificial Intelligence (CONSORT-AI) are the international standards for AI system clinical trials, enhancing the integrity and transparency [119]. SPIRIT-AI is an extension of the clinical trial protocol guide SPIRIT 2013 with 15 new entries; And the CONSORT-AI is an extension of the clinical trial reporting guide CONSORT 2010 with 14 new entries [120]. These standards have provided detailed descriptions of AI interventions, instructions, skills, and the integration environment required for use, inputs and outputs, human-computer interaction details, and provision of error case studies. However, SPIRIT-AI and CONSORT-AI mainly focus on supervised learning, with limited guidance on handling unsupervised and self-supervised learning. Additionally, these standards are predominantly image-based and currently lack constructive guidance for speech and text types.

## 7. Obstacles and Solutions for Clinic Implementation of AI in Pathology

Although the data preparation methods mentioned above have led to the development of robust AI systems that exhibit effectiveness and accuracy in laboratory settings [60], significant obstacles persist, impeding the replication of high performance AI in clinical practice [121].

Firstly, the number of WSIs in public datasets is notably limited, containing only typical manifestations of diseases. These images are rigorously stain-normalized and carefully confirmed by several experienced pathologists [122]. However, they are deliberately selected to represent specific characteristics, failing to cover the full spectrum of biological and morphological variations seen in clinical cases. Consequently, AI may struggle to identify diverse disease morphologies encountered in clinical practice. The datasets collected from the clinic can be as many as tens of thousands of WSIs, so models can often achieve better performance. However, these datasets cannot contain enough disease types or subtypes which are rare [123], so the performance of AI degrades rapidly while these subtypes appear.

Secondly, there are great differences in slide production and digitization protocols across multi-centers, as well as variations in color among images, present significant challenges. Issues such as bubbles, tissue folding, and image blurring may reduce image quality and cause a decrease in the performance of AI. Although normalization techniques such as color correction offer some relief, they may require retraining when AI is deployed in new centers.

Thirdly, even when a sufficient number of WSIs are available, the number of annotation is still limited, especially for fine annotation of rare diseases, remains a bottleneck for clinical AI performance [124]. Weak and sparse annotations can expedite annotation speed but fall short in addressing the challenge of insufficient data and lack of representative samples. Although GAN can augment data, they face difficulty accurately generating cases not present in the training set. The unsupervised pre-training and semi-supervised learning may reduce annotation requirements, but their performance rely on the coverage of typical disease morphology [125].

Based on the discussion above, the main obstacle of AI used in clinic is the dataset such as lack of representative samples that cannot cover all morphologies. Due to the lack of standardization, the differences in production protocols and digitization in other centers have resulted in a decline in the performance of AIP trained on one center. The third obstacle is that the number of annotations is too small. There are three strategies to hopefully overcome the obstacles.

### 7.1. Develop Digital Pathology for Clinical Diagnosis

In contemporary medical practice, a significant portion of samples still exists in the form of slides due to limited digitalization. As previously discussed, the digitization of these slides for data preparation in Pathological Artificial Intelligence is a time-consuming endeavor. This is primarily because the current diagnosis is based on slides rather than digital images. Despite the increasing use of high-resolution pathological scanners, the sheer number of pixels in Whole Slide Images (WSI) leads to exorbitant storage and scanning time costs. Consequently, the high cost of slide digitization constrains the clinical application of digital pathology, with slide-based diagnosis remaining the norm across most medical institutions [126].

The shift to digital pathology in clinical diagnosis, where all the samples are images and not slides, holds the potential to significantly increase the number of available samples while alleviating the lack of representative samples. The key to fostering the growth of digital pathology is to reduce the cost of digitization, including storage and scanning time. An innovative approach involves low-resolution digitization, where the slides are initially scanned with a 5× lens, storing 5× images that are later enhanced to 40× images using super-resolution techniques. This approach not only reduces digitization costs to a fraction (1/64) but also maintains diagnostic accuracy, demonstrating promise in addressing the high cost of slide digitization. By promoting digital pathology, this method facilitates the accumulation of sufficient high-quality data for training clinical-grade AI [127].

### 7.2. Standardization for Slide Production and Image Digitization

There are many differences in the production protocol and digitization equipment across different centers which results in variations between images used for training AI and those encountered in clinical practice. The slide preparation and digitization standardization can reduce the differences and improve the cross-center performance. Firstly, production protocols should undergo standardization, encompassing factors such as chemical concentrations and well-defined quality standards for slides. Secondly, the resolution, sharpness or color fidelity of pathological scanners must be meticulously evaluated, guiding the formulation of digitization guidelines and industry standards for scanners. Additionally, newly generated WSIs should undergo automated quality checks before entering AI workflows. Therefore, the industry standards of slide production and image digitization are crucial in harmonizing clinical data, with quality checks contributing to consistent AI performance in clinical practice.

### 7.3. WSI-Level Annotation and Weakly Supervised Learning

The time-intensive nature of pixel-level or patch-level annotations often results in an insufficient amount of labeled data for training AI in pathology. With the promotion of digital pathology, as discussed in Section 7.1, it is not difficult to obtain enough WSI for training. However, there is still a major obstacle in labeling a large number of pixels or patches. It is worth noting that WSI-level annotation is inherently available due to pathology diagnoses conducted on WSIs. Weakly supervised learning approaches, such as multi-instance learning do not mandate precise location annotations, rendering WSI-level annotation a viable alternative. Several AI models leveraging weakly supervised learning based on WSIs have demonstrated promise. The WSIs were coded into bags, and contextual information from different instances was harnessed to achieve impressive results, including an AUC of 0.99 for lung adenocarcinoma classification and an AUC of 0.73 for lymph node metastasis prediction [110]. A multi-instance learning approach based on clustering and attention mechanisms was proposed to improve data validity and achieved excellent performance in cancer classification [109]. Recently, the WSI- based AI focusing on three cancers obtained from the Anatomical Pathology Laboratory Information System (LIS) demonstrated an AUC exceeding 0.98 [23].

While AI performance based on WSI-level weak annotation may not yet rival that of fine annotation, it is poised to adapt to the evolving landscape of digital pathology, where annotation complexities are expected to diminish. It is believed that the combination of sufficient annotated data from clinic and weakly supervised learning at WSI level holds significant promise for developing the clinical-grade AIP.

## 8. Conclusions

Data serves as the cornerstone of Artificial Intelligence (AI) in Pathology, and effective data preparation is an indispensable step in achieving high-performance AI model. In this review, we underscore the pivotal role of high-quality data in the field of AI, conducting a detailed analysis of how the data preparation process influences data quality, such as the acquisition and preprocessing of Whole Slide Images (WSIs). Given the demand for large datasets, employing weak annotation, patch-level annotation, or WSI-level annotation can reduce the workload while yielding results closely approaching fine annotation. Dataset partitioning also holds significance. Ensuring the independence of different datasets and maintaining a balanced distribution of different disease subtypes is essential to ensure model performance stability.

To address the issue of insufficient available data, hospitals and medical institutions should vigorously promote digital pathology, converting more pathological glass slides into digital images for enhanced inter-institutional collaboration and data sharing. However, it is imperative to be mindful of privacy and ethical considerations when conducting research on pathological AI, adhering to relevant regulations and ethical guidelines to ensure the confidentiality and security of patient data.

Future research and practice should continuously advance pathology digitization, explore novel methods to enhance data quality, establish robust privacy protection mechanisms, and adhere to ethical standards. These efforts will further propel the field, facilitating the broader application of pathological AI in clinical practice and providing superior solutions for healthcare.

Finally, it should be noted that while we conducted a comprehensive search on the PubMed database, there are noteworthy works, particularly in the rapidly evolving field of AI in pathology, that may not be indexed on PubMed for various reasons. Nonetheless, we genuinely believe that the 118 papers selected can provide a comprehensive and robust foundation to support the above conclusions.

## Figures and Tables

**Table 1:** Public datasets for AI in pathology.

Dataset	Number	Disease Type	Reference
PatchCamelyon	400 WSI	lymph nodes	[43]
GlaS	165WSI	Stage T3 or T4 colorectal adenocarcinoma	[44]
LUAD-HistoSeg	-	lung cancer	[45]
Camelyon16 Dataset	400 WSI	lymph nodes	[41]
Colorectal cancer	10 WSI and 5000 patches	bowel cancer	[46]
PAIP	100WSI	liver	[47]

## Supplementary Materials

The following supporting information can be downloaded at: https://www.mdpi.com/article/10.3390/diagnostics13193115/s1, Table S1: The detailed information of the datasets in reviewed papers.
