# Supplementary material for: Preparing Data for Artificial Intelligence in Pathology with Clinical-Grade Performance
Source: Diagnostics (Basel). 2023 Oct 3;13(19):3115. doi: 10.3390/diagnostics13193115 (PMC10572440; doi:10.3390/diagnostics13193115)
Supplement: Supplementary file 1 [file diagnostics-13-03115-s001.zip › diagnostics-2614561-supplementary.pdf]

Table S1: The detailed information of the datasets in reviewed papers.

Task	Data set	Cancer type	Scanner	Patch size	Annotation method	Pre-processing method
**Classification** [7]	227 WSIs	High-grade extrauterine serous carcinoma	Pannoramic SCAN 0.22 µm/pixel	/	Pixel-level annotation	/
**Classification** [8]	3212 WSIs	Gastric	Hamamatsu;Roche; KFBio; 20X	320 × 320	Pixel-level annotation	Data augmentation
**Classification** [9]	896 WSIs	Gastric	Nanozoomer 2.0-HT	224x224	Weak annotation(box)	Data augmentation

**Classification** [10]	4712 WSIs	Prostate	Pannoramic Flash II 250 (3DHistech, Hungary) scanner at a pixel resolution of 0.24µm	/	WSI-level	/
**Gleason grading** [11]	641 WSIs	Prostate	NanoZoomer-XR	224x224	Weak annotation	Data augmentation (rotations, flipping and color jittering)
**Classification** [19]	3470 WSIs	Breast	Carl Zeiss slide scanner, 40X resolution (0.249 microns per pixel)	224x224	WSI-level	Data augmentation (rotation, color spectrum augmentation1, color normalization)
**Classification** [23]	44,732 WSIs	Breast	Leica Aperio AT2 scanners at 20X equivalent magnification 0.5 µm per pixel	/	Weak annotation	/

**Classification** [25]	13,111 WSIs	Colorectal	KF-PRO-005 scanner 20X	300x300	Fine annotation	data augmentation (image zoom, flip, color change)
**Classification** [26]	7003 WSIs	Lung	Aperio scanner in either 20X (0.50 µm per pixel) or 40X magnification (0.25 µm per pixel)	224x224	WSI-level	Data augmentation (flipping,translation,rotation, and color augmentations)
**Gleason grading** [27]	1243 WSIs	Prostate	3DHistech Pannoramic Flash II 250 20X(0.24µm per pixel)	/	WSI-level	/
**Classification** [30]	1584 WSIs	Pan-Renal Cell Carcinoma	/	224x224	WSI-level	data augmentation techniques (random horizontal flip and random Crop)

**Classification** [31]	193 WSIs	Oral squamous cell carcinoma	Omnyx Integrated Digital Pathology system,40X 0.275μm per pixel	128x128	Weak annotation	data augmentation (random rotation and random flipping)
**Classification** [33]	9366 WSIs	Gastric	Jiangfeng scanner at 40X,0.2513μm per pixel; Hamamatsu scanner at 40X,0.2206μm per pixel	768x768	Fine annotation	Data standardization
**Classification** [34]	128 images	Prostate	NanoZoomer digital slide scanner 0.23μm per pixel	1024x1024	Fine annotation	Data augmentation (rotation, flip, normalization)
**Segmentation** [45]	54 WSIs	Lung	/	224x224	Weak annotation	/

**Classification** [49]	2323 WSIs	Lung	/	224x224	Fine annotation	Data augmentation (flipping, rotations, and shifts in brightness, contrast, hue, and saturation)
**Classification** [58]	717 WSIs	Prostate	Philips Ultra Fast Scanner, Panoramic 250 Flash II scanner, Olympus VS120-S5	/	Fine annotation	Data augmentation, Rescanning, style normalization
**Classification** [59]	100WSIs	Liver	20X,15X	/	Weak annotation	/
**Classification** [61]	422 WSIs	Lung	Leica Aperio whole-slide scanner at 20x	224x224	Fine annotation	Color normalization, data augmentation (rotation, flip)

**Classification** [66]	148 WSIs	Translocation renal cell carcinoma	Leica Aperio scanner at ×40	2000x2000	/	Color normalization
**Gleason grading** [71]	/	Lymph node, prostate	Ventana scanner of 0.25μm per pixel;Hamamatsu scanner of 0.23 μm per pixel	/	Fine annotation	Stain normalization
**Prognostic** [86]	/	Gliomas	/	1024x1024	/	Data augmentation (GAN)
**Classification** [90]	2647 patches	Cervical	/	256x128	Weak annotation	Data augmentation (GAN)

**Detection** [94]	3000 patches	Colorectal	Aperio ScanScope XT at 40X of 0.061µm per pixel	256x256	Fine annotation	/
**Classification** [95]	284 WSIs	Lung	BX53, Olympus Corporation at 40X	256x256	/	Data augmentation (rotation, flipping, and color correction,)
**Segmentation** [96]	1400 images	Gastric	/	512x512	Weak annotation	data augmented (horizontal flips, rotations, random crops and hue saturation value shifts)
**Classification** [98]	905 WSIs	Stomach	/	256x256	Fine annotation	Eliminate noises

**Detection** [99]	45 WSIs	Breast	Leica Aperio AT2 whole slide scanner at 20X of 0.5 µm per pixel.	64x64	Weak annotation	Data augmentation
**Classification** [100]	3554 WSIs	Lung	20X	512x512	Fine annotation	Data augmentation (flip, translation, color shift)
**Classification** [106]	5846 images	Melanoma	/	/	Box annotation	Data augmentation (horizontal flip, random distort, rotations, crop, zoom)
**Classification** [108]	400 WSI and 993WSIs	Kidney, lung	/	256x256	Fine annotation	/

**Classification** [111]	508 WSIs	Colorectal	Aperio AT2, Leica Biosystems at 40× resolution (0.25-μm pixel−1)	224x224	Fine annotation	Data augmentation (rotation, flip, color jitter on the brightness, contrast, saturation and hue of each image)
